# Malnutrition Imparts Worse Outcomes in Patients With Diverticulitis: A Nationwide Inpatient Sample Database Study

**DOI:** 10.7759/cureus.26973

**Published:** 2022-07-18

**Authors:** Amjad Shaikh, Ayham Khrais, Alexander Le, Sushil Ahlawat

**Affiliations:** 1 Internal Medicine, Rutgers University New Jersey Medical School, Newark, USA; 2 Gastroenterology and Hepatology, Rutgers University New Jersey Medical School, Newark, USA

**Keywords:** sepsis, internal medicine, diverticulitis, malnutrition, gastroenterology

## Abstract

Introduction

Studies show that malnutrition can lead to worsening morbidity and mortality in patients. However, to our knowledge, no large database study has been conducted describing the effects of malnutrition in patients with diverticulitis. In this article, we aim to assess the impact of pre-existing malnutrition on outcomes of patients admitted for diverticulitis.

Methods

Data between 2008 and 2014 from the Nationwide Inpatient Sample database were extracted. Inclusion criteria for both groups included patients with a primary diagnosis of diverticulitis using the International Classification of Diseases, Ninth Revision codes. Exclusion criteria included all patients less than 18 years of age. The test group consisted of patients with a primary diagnosis of diverticulitis and a concurrent diagnosis of malnutrition. In-hospital mortality, length of stay, total cost, and complications, including various forms of sepsis, perforation, bleeding, and GI bleeding, were compared between the two groups. Univariate and multivariate analyses were used to generate odds ratios. Multivariate analysis included age, sex, race, income quartile, and calculated Elixhauser scores. Elixhauser comorbidity scores predicting mortality and readmission were calculated based on weighted scores from 29 different comorbidities. Scores were compared between the two groups using univariate analysis.

Results

There were a total of 1,520,919 patients in the study, of which 427,679 (2.8%) had a pre-existing diagnosis of malnutrition. On univariate analysis, there was a significant increase in mortality in patients with malnutrition (OR: 10.2, p < 0.01). Additionally, patients with malnutrition appeared to have longer lengths of stay (mean: 12.9, p < 0.01) and greater cost of hospitalization (mean: 194436.82, p < 0.01). Patients with malnutrition had greater rates of sepsis events (OR: 12.0, p < 0.01), perforation (OR: 2.8, p < 0.01), and GI bleed (OR: 1.84, p < 0.01). On multivariate analysis, malnutrition appeared to significantly increase mortality (OR: 3.3, p < 0.01).

Discussion

Patients who present with diverticulitis with malnutrition appear to have significantly worse outcomes. We hypothesize that malnutrition leads to a shift in the gut microbiota, resulting in increased inflammation. As a result, these patients may have an increased risk of worse outcomes, such as sepsis and death. Addressing nutrition in patients with diverticulosis or those with a history of diverticulitis may improve outcomes.

This abstract was previously presented at the Digestive Disease Week Conference on May 22, 2022. Abstracts accepted at the conference were published in supplements of the journals Gastroenterology and GIE: Gastrointestinal Endoscopy.

## Introduction

Diverticulosis involves the formation of small pouches at points of weakness within the wall of the colon, specifically at the site where the muscularis mucosae are traversed by vasa recta [1-3]. Increased intraluminal pressure at this specific location results in the formation of diverticula. Therefore, patients on low fiber diets are predisposed to diverticular disease [3]. The strength of colonic contraction is the strongest within the sigmoid and descending colons, as such generation of intraluminal pressure is the greatest in these regions. Therefore, diverticular disease occurs most commonly within the sigmoid and descending colons. Diverticulitis is inflammation of colonic diverticula and is subdivided into un-complex diverticulitis, denoting colonic inflammation only, and complex diverticulitis, which may involve the development of an abscess, fistula, colonic perforation, or bleeding [2]. It is thought to be caused by microperforation of existing diverticula as a result of increased intraluminal pressure. Therefore, factors that either increase intracolonic pressure or compromise the structural integrity of the colonic wall can predispose patients to diverticulitis. Such factors include age, obesity, smoking, low exercise rates, and diet (intake of low fiber and excess red meat) [2,4]. Diagnosis is via a combination of symptomatology (including lower quadrant abdominal pain), laboratory evaluation (such as leukocytosis and elevated inflammatory markers), and imaging (CT scan) [2]. With increasing yearly prevalence, diverticulitis affects 180 per 100,000 individuals annually and is becoming a more relevant source of significant mortality and morbidity in the population of the United States [4,5]. Efforts to further understand risk factors for diverticulitis can aid in the comprehension of this trend of increasing prevalence and how it can be combated. While some risk factors, including age, are non-modifiable, others, including diet, can be altered to reduce one’s predilection for this disease.

Malnutrition describes the deprivation of the nutrients, vitamins, and minerals required for the body to maintain tissue function and health [6]. Malnutrition is commonly thought of in those who are undernourished: those who do not consume enough nutrients to meet their body’s daily requirements. This can be due to decreased oral intake, malignancy, chronic alcohol use, or decreased intestinal absorption [7]. Alternatively, malnutrition can be associated with overnourishment; individuals who eat excessively and who do not exercise sufficiently can have difficulty maintaining organ function [7].

Limited research endeavors have explored the link between nutritional status and diverticulitis. Among those projects, a smaller number have established a positive correlation between pre-existing malnutrition and the onset of acute diverticulitis [8]. Furthermore, malnourished patients hospitalized for diverticulitis were also found to have longer hospital stays [8]. Diverticulitis itself has also been shown to worsen pre-existing malnutrition, as the pain can result in oral intake intolerance and anorexia [9]. Therefore, nutritional supplementation may prove beneficial to those hospitalized for diverticulitis. We aim to further explore this link to better characterize the outcomes of diverticulitis among those who are malnourished.

This article was previously presented as a poster at the Digestive Disease Week Conference on May 22, 2022. Abstracts accepted at the conference were published in supplements of the journals Gastroenterology and GIE: Gastrointestinal Endoscopy.

## Materials and methods

Data/source population

Data between 2008 and 2014 from the Nationwide Inpatient Sample (NIS) were extracted. Inclusion criteria for both groups encompassed patients with a primary diagnosis of acute diverticulitis using the International Classification of Diseases, Ninth Revision (ICD-9) codes, and those aged 18 years old or older.

The experimental group was comprised of patients with a primary diagnosis of acute diverticulitis and a second diagnosis of malnutrition. The following confounders were controlled for upon data analysis: age, gender, race, and median household income.

Statistical analysis

Categorical and continuous variables were analyzed via chi-square analysis and Student's t-test, respectively. In-hospital mortality was compared between the experimental and control groups. Elixhauser Comorbidity Index (ECI) scores, designed to predict mortality and readmission rates, were calculated based on weighted scores from 29 different comorbidities. ECI scores were compared across both groups via Student's t-test. Multiple comorbidities were also assessed between both groups. Multivariate analyses using binary logistic regression were conducted with death as the primary outcome. ECIs were generated using SAS OnDemand for Academics (SAS Institute, Cary, NC) and Statistical Package for the Social Sciences (SPSS) version 28.0.0 (IBM Corporation, Armonk, NY). Multivariate analysis factoring in age, sex, ethnicity, income status, and ECI scores were used to further characterize and assess the effects of malnutrition on mortality.

## Results

In terms of patient characteristics, those with malnutrition were older (68.86 years versus 59.29 years). Most patients in both groups were females; however, those with malnutrition had a greater proportion of females (66.8% versus 57.3) as opposed to those without malnutrition (Table 1). The majority of both groups were Caucasian (82.4% in malnourished patients versus 77.3% in non-malnourished patients); however, the second most common race in the malnourished group was Black (7.2%), while that of the non-malnourished group was Hispanic (10.8%) (Table 1).

**Table 1:** Patient demographics

		No malnutrition (n = 1,478,240)	Malnutrition (n = 42,679)
Age (SD)		59.29 years (15.5)	68.85 years (14.48)
Sex	Male (%)	629,466 (42.7)	14,176 (33.2)
	Female (%)	846,301 (57.3)	28,479 (66.8)
Race	Caucasian (%)	1,034,748 (77.3)	31,843 (82.4)
	Black (%)	107,079 (8.0)	2,788 (7.2)
	Hispanic (%)	144,695 (10.8)	2,551 (6.6)
	Asian/Pacific Islander (%)	11,976 (0.9)	383 (1.0)
	Native American (%)	6,089 (0.5)	151 (0.4)
	White (%)	34,666 (2.6)	910 (2.4)

Patients with malnutrition hospitalized with acute diverticulitis had greater mortality than those without malnutrition hospitalized for the same issue (3.9% versus 0.4%) (Table 2). Complication rates stemming from acute diverticulitis were significantly elevated in the malnutrition group, including colonic perforation, fistula formation, gastrointestinal bleeding, respiratory failure, sepsis, abscess formation, ileus, and obstruction (Table 2).

**Table 2:** Rates of mortality and patient complications

	No malnutrition (n= 1,478,240)	Malnutrition (n = 42,679)	Odds ratio (CI)	P-value
Died (%)	5,908 (0.4)	1,684 (3.9)	10.24 (9.69-10.82)	<0.01
Perforation (%)	9,368 (0.6)	754 (1.8)	2.82 (2.62-3.04)	<0.01
Fistula (%)	9,541 (0.6)	1,133 (2.7)	4.198 (3.94-4.47)	<0.01
Bleed (%)	9,539 (0.6)	504 (1.2)	1.84 (1.68-2.01)	<0.01
Respiratory failure (%)	39,288 (2.6)	3,391 (24.3)	11.98 (11.51-12.47)	<0.01
Sepsis event (%)	17,761 (1.2)	5,452 (12.8)	12.04 (11.66-12.44)	<0.01
Abscess (%)	29,946 (2.3%)	12,733 (5.8)	2.61 (2.56-2.67)	<0.01
Ileus (%)	34,599 (2.4)	8,081 (9.9)	4.47 (4.35-4.58)	<0.01
Obstruction (%)	40,403 (2.7)	2,277 (9.9)	3.977 (3.80-4.16)	<0.01

ECI mortality (16.87 versus 2.33) and readmission (27.77 versus 7.9) rates were higher in those with malnutrition (Table 3). Length of stay (12.89 days versus 4.64 days) and total charges ($32,157.93 versus $194,436.82) were significantly more elevated in malnourished patients (Table 3).

**Table 3:** Analysis of length of stay, total charges, and Elixhauser indices

	No malnutrition	Malnutrition
Length of stay (SD)	4.64 days (4.14)	12.89 days (11.38)
Total charges (SD)	$32,157.93 ($42,128.17)	$194,436.82 ($130,389.53)
Elixhauser mortality (SD)	2.33 (6.79)	16.87 (9.47)
Elixhauser readmit (SD)	7.90 (10.75)	27.77 (12.30)

**Figure 1:**
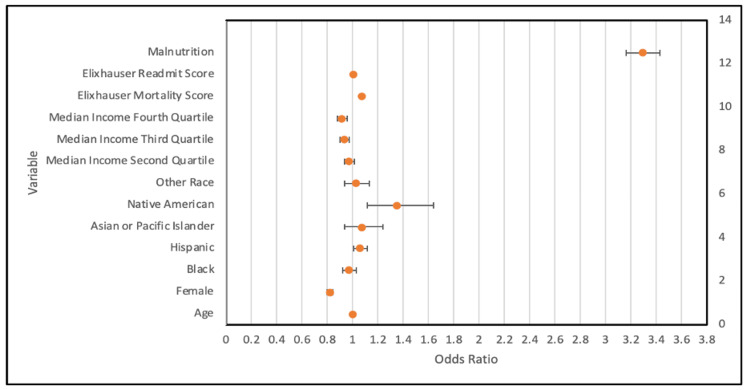
Multivariate analysis of mortality

On multivariate analysis, malnutrition had a significant effect on mortality (odds ratio: 3.294, Cl: 3.161-3.431). Additionally, age, female status, Hispanic, Native American, third and fourth quartile income status, and ECI scores were all significant (Table 4, Figure 1).

**Table 4:** Odds ratios generated from multivariate analysis

	Odds ratio	CI lower 95%	CI upper 95%
Age	1.003	1.002	1.004
Female	0.822	0.798	0.847
Black	0.974	0.922	1.028
Hispanic	1.062	1.01	1.116
Asian or Pacific Islander	1.077	0.935	1.241
Native American	1.351	1.115	1.637
Other race	1.03	0.938	1.131
Median income second quartile	0.973	0.935	1.012
Median income third quartile	0.936	0.899	0.974
Median income fourth quartile	0.919	0.883	0.958
Elixhauser mortality score	1.076	1.073	1.078
Elixhauser readmit score	1.011	1.009	1.012
Malnutrition	3.294	3.161	3.431

## Discussion

Adequate nutritional intake is necessary for the maintenance of cell homeostasis and tissue function. Proper nourishment depends on the intake of a variety of micronutrients such as vitamins and metals, as well as macronutrients such as proteins, lipids, and carbohydrates [6,7,10-13]. Poor nutritional status is not only a result of inadequate ingestion of these components but also from overconsumption, defects in intestinal absorption, or issues with metabolism. Therefore, determining the cause of existing malnutrition can be difficult as it may be multifactorial. Furthermore, properly assessing one’s nutritional status may also be difficult. Laboratory values such as low albumin and pre-albumin have been utilized as indicators of malnutrition; however, abnormalities in these values can be caused by a diverse array of pathology, including liver cirrhosis and kidney disease [13]. Alternatively, physical exam findings, including BMI, are utilized to preliminarily identify nutritional status [13]. Such physical findings may be overt as in cachexia (decreased muscle mass, loss of subcutaneous fat, and decreased muscle strength) or covert (brittle nails, hair loss, and fatigue). Therefore, full determination of a patient’s nutritional status requires a thorough history, including a nutrition overview and physical and laboratory assessment.

Identifying malnutrition is vital because of its association with poor inpatient outcomes, including prolonged length of stay, inpatient mortality, mortality within 180 days post-discharge, and higher rates of readmission [10,14,15]. Furthermore, total charges related to the hospital stay have been shown to be higher in malnourished patients [14]. Therefore, nutritional status represents a potentially clinically relevant prognostic indicator that could shed light on inpatient and post-discharge outcomes in hospitalized individuals. Despite this clear trend of worsening outcomes with poor nutritional status, few investigations show improvement in diverticulitis outcomes with nutritional supplementation. There is some evidence demonstrating decreased mortality after discharge in malnourished COPD patients who used high protein supplementation [16]. On the other hand, the use of similar supplementation in other malnourished populations demonstrated no difference in post-discharge mortality and readmission rates; however, some improvement in inpatient mortality was observed [17,18].

Once malnutrition is identified, ascertaining an underlying etiology may assist in developing an adequate treatment plan. Acute diverticulitis is one such cause that is intricately linked with nutritional status. It is an uncommonly recognized cause of nutritional deficiency, for it most often occurs in overweight patients [8]. However, as established, overnourishment is associated with malnutrition and is often overlooked in overweight and obese patients due to the common misconception that poor nutritional status is only associated with low BMI [7,8]. Furthermore, malnourishment has been associated with increased rates of acute diverticulitis, as well as prolonged hospital stays [8,19]. This is likely due to a combination of a nutritional intake consisting of significant red meat and low fiber [19]. This diet will slow colonic transit, increasing intraluminal pressure and resulting in microperforation. In effect, malnutrition may cause acute diverticulitis, which begets worsening malnutrition.

Various diets have also been hypothesized to affect the composition of gut microbiota [19]. For example, diets with a large amount of red meat have been associated with an increased prevalence of *Enterobacteriaceae* within the gut microbiome [19]. A preponderance of this bacterial species, as well as *Bacteroides* and *Streptococcus*, has been associated with diverticular disease [20]. Furthermore, a link between microbiome composition and colonic inflammation has been established, with some cases demonstrating the onset of diverticulitis after changes to the colonic microbiome [21]. The pathophysiology of this correlation is still unclear; however, a link between diet, microbiome, and colonic inflammation exists and requires further exploration.

In our analysis, we found that malnourished patients had longer hospital stays, greater hospital charges, and higher rates of readmissions after initial discharge. Furthermore, we found that malnutrition in patients hospitalized for acute diverticulitis was significantly associated with increased inpatient mortality, as well as rates of complications, including bowel perforation, fistula formation, gastrointestinal bleeding, intra-abdominal abscess formation, ileus, colonic obstruction, respiratory failure, and sepsis. Increased rates of complications in the setting of poor nutritional status are likely multifactorial. As previously established, slowed colonic transit results in the buildup of intraluminal colonic pressure, while poor nutritional diets can increase the prevalence of pro-inflammatory bacteria within the microbiome [18-21]. These changes can contribute to worsening intraluminal microperforation and inflammation, thereby increasing rates of diverticulitis and worsening outcomes.

This study is limited by our use of the NIS database, which utilizes ICD-9 codes to identify diagnoses. As malnutrition is difficult to clinically diagnose, it may be underreported and therefore underrepresented in our sample size. Furthermore, malnutrition is often diagnosed either by physical exam or laboratory assessment; however, multiple diagnostic tools also exist to arrive at this diagnosis [13,22,23]. Despite this, the NIS database does not allow for the stratification of nutritional status due to its coding structure. While hypoalbuminemia is often used to diagnose malnutrition, albumin may be low due to alternate causes in response to an acute inflammatory state, as a result of increased vascular permeability and resultant shifting of albumin to the extravascular space [22]. Diagnosis of malnutrition based solely on hypoalbuminemia may be false, and subsequent coding for malnutrition may result in an overestimation of this diagnosis. Patients admitted for acute diverticulitis, an acute inflammatory state, may have falsely low levels of albumin, thereby resulting in a misdiagnosis of malnutrition, causing possible overestimation of nutritional deficiency in this patient population.

## Conclusions

Malnutrition has been associated with worse outcomes in those hospitalized for acute diverticulitis, including mortality, complication rates, length of stay, and hospital costs. Poor nutritional status should be identified to better stratify those at increased risk of complications; however, more objective methods of diagnosing malnutrition should be identified. Potentially subjective lab findings, such as albumin, which can be altered for multiple reasons, should not be used in all patients to identify their nutritional status. Objective tools that are specific to diagnosing malnutrition and can stratify the degree would help determine if its association with diverticular disease is step-wise in nature. There is no established benefit to correcting poor nutritional status in this patient population. Further studies are required to determine if nutritional supplementation would benefit malnourished patients hospitalized with diverticulitis.
